# Translocator Protein 18 kDa (TSPO) Is Regulated in White and Brown Adipose Tissue by Obesity

**DOI:** 10.1371/journal.pone.0079980

**Published:** 2013-11-18

**Authors:** Misty M. Thompson, H. Charles Manning, Kate L. J. Ellacott

**Affiliations:** 1 Department of Molecular Physiology and Biophysics, Vanderbilt University Medical Center, Nashville, Tennessee, United States of America; 2 Department of Radiology and Vanderbilt Institute of Imaging Science, Vanderbilt University Medical Center, Nashville, Tennessee, United States of America; State University of Rio de Janeiro, Biomedical Center, Institute of Biology, Brazil

## Abstract

Translocator protein 18 kDa (TSPO) is an outer-mitochondrial membrane transporter which has many functions including participation in the mitochondrial permeability transition pore, regulation of reactive oxygen species (ROS), production of cellular energy, and is the rate-limiting step in the uptake of cholesterol. TSPO expression is dysregulated during disease pathologies involving changes in tissue energy demands such as cancer, and is up-regulated in activated macrophages during the inflammatory response. Obesity is associated with decreased energy expenditure, mitochondrial dysfunction, and chronic low-grade inflammation which collectively contribute to the development of the Metabolic Syndrome. Therefore, we hypothesized that dysregulation of TSPO in adipose tissue may be a feature of disease pathology in obesity. Radioligand binding studies revealed a significant reduction in TSPO ligand binding sites in mitochondrial extracts from both white (WAT) and brown adipose tissue (BAT) in mouse models of obesity (diet-induced and genetic) compared to control animals. We also confirmed a reduction in TSPO gene expression in whole tissue extracts from WAT and BAT. Immunohistochemistry in WAT confirmed TSPO expression in adipocytes but also revealed high-levels of TSPO expression in WAT macrophages in obese animals. No changes in TSPO expression were observed in WAT or BAT after a 17 hour fast or 4 hour cold exposure. Treatment of mice with the TSPO ligand PK11195 resulted in regulation of metabolic genes in WAT. Together, these results suggest a potential role for TSPO in mediating adipose tissue homeostasis.

## Introduction

As of 2010 more than one-third of Americans were defined as obese [1]. Obesity is one of the leading risk factors for developing Metabolic Syndrome which comprises dangerous co-morbidities including insulin resistance, hypertension, and dyslipidemia. It has been well documented that chronic low-grade inflammation and decreases in energy expenditure provide a signaling cascade through which these symptoms emerge [2], [3]. In particular, adipose tissue is one of the principal sites of inflammation and mitochondrial dysfunction which promotes insulin insensitivity and metabolic dysregulation downstream of obesity [4], [5].

TSPO, or translocator protein, is an 18 kDa outer-mitochondrial membrane transporter which has many functions including participation in the mitochondrial permeability transition pore, regulation of reactive oxygen species (ROS) production, apoptosis, production of cellular energy, and is the rate-limiting step in the uptake of cholesterol, and thus, steroidogenesis [6]. Global knockout of TSPO protein in mice is embryonic lethal suggesting it is essential for mitochondrial function [7]. TSPO expression is highly up-regulated in activated microglia in the CNS as well as macrophages in the periphery during the inflammatory response [8], [9]. Furthermore, TSPO is also dysregulated during disease pathologies such as cancer which involve changes in cellular energy demands [6]. This has led to the emergence of positron emission tomography (PET) using TSPO ligands as a reliable biomarker used for noninvasive imaging in disease pathologies such as Alzheimer’s disease and cancer in both animals and humans [10], [11].

While TSPO expression is highest in steroidogenic tissues, several studies in rodents have revealed changes in TSPO expression in metabolic tissues such as liver, heart, and brown adipose tissue (BAT) [12], [13], [14]. Studies in 3T3-L1 and SW872 preadipocyte cell lines have shown that TSPO expression is increased during adipocyte differentiation but then decreases during the maturation phase [15], [16]. A study of white adipose tissue (WAT) in rats also suggested that TSPO can be up-regulated in response to a stressful stimulus [17]. These studies, coupled with the finding that rats provided a high-fat, high-cholesterol (HFHC) diet have decreased TSPO binding capacity in the liver and heart [12], prompted us to determine whether obesity also regulates TSPO expression in WAT and BAT. Given the roles of TSPO in mitochondrial function and its up-regulation in activated macrophages, combined with the known effects of obesity on mitochondrial dysfunction and inflammation in mice and humans, we hypothesized that TSPO levels would be dysregulated in adipose tissue by obesity. Herein, we provide data from two mouse models, diet-induced obese (DIO) and melanocortin 4 receptor knockout mice (MC4R^−/−^; [18]), which reveal that TSPO gene expression and protein density decrease in WAT and BAT as a function of obesity.

## Materials and Methods

### Ethics Statement

All experiments were approved by and carried out in strict accordance with the guidelines of the Institutional Animal Care and Use Committee (IACUC) at Vanderbilt University Medical Center (Protocol Number M/09/034). At the time of sacrifice, animals were euthanized with an overdose of isoflurane to minimize suffering followed by decapitation.

### Animal Studies

The animals used in the experiments were male C57BL6/J (Stock no. 000664, Jackson Laboratory, Bar Harbor, ME) or male melanocortin 4 receptor knockout mice (MC4R^−/−^) on a C57BL6/J genetic background, obtained from a colony maintained at Vanderbilt University which were derived from the original founders [18]. Animals were housed at 21±2°C under a 12 hour light/dark cycle in a sterile barrier facility. For DIO studies, beginning at 8 weeks of age, mice (n = 7–8) were provided *ad libitum* high-fat diet (HFD; 60% kcal from fat, Cat. no. D12532, Research diets Inc., New Brunswick NJ) or maintained on standard laboratory chow (13% kcal from fat, PicoLab Rodent Diet 20, LabDiet, PMI Nutrition International, St. Louis, MO) for 19 weeks, and body weights were recorded weekly. Male MC4R^−/−^ and wild-type littermate controls (MC4R^+/+^; n = 6/group) were maintained on *ad libitum* standard chow until 34 weeks of age. At the time of euthanasia, body composition was determined for all mice using the Bruker nuclear magnetic resonance (NMR) imaging unit in the Vanderbilt Mouse Metabolic Phenotyping Center (MMPC) which revealed comparable increases in fat mass between the two obesity models compared to their respective controls (Table 1). After deep euthanasia to minimize suffering, animals were rapidly perfused transcardially with 0.9% saline and the two pads of epididymal WAT and interscapular BAT removed, frozen on dry ice, and then stored at −80°C.

**Table 1:** Body composition of diet-induced obese and MC4R^−/−^ mice utilized in radioligand binding studies compared to their respective lean controls.

	Lean	Obese	*p*	MC4R^+/+^	MC4R^−/−^	*p*
n	7	8		6	6	
Body weight (g)	30.20±0.34	46.28±0.73	<0.0001	26.42±0.52	47.97±1.58	<0.0001
Muscle mass (g)	23.50±0.42	26.84±0.56	0.0005	20.27±0.18	28.34±0.72	<0.0001
Fat mass (g)	2.13±0.34	15.80±0.51	<0.0001	2.25±0.26	15.57±0.85	<0.0001

Data are presented as mean ± standard error of the mean (s.e.m.). Statistical significance was taken and *p*<0.05 as determined by unpaired t-test. g = grams.

For the fasting experiment, individually housed 13–20 week old male C57BL6/J mice were euthanized as described above after 17 hours of fasting with control animals fed ad libitum (n = 13–14/group). For cold exposure studies, 21–25 week old male C57BL6/J mice were individually housed for 3 days prior to undergoing 4 hours of exposure to temperatures between 4–8°C in a refrigerated incubator (Powers Scientific, Inc.; Pipersville, PA) or held at room temperature (22°C) as a control (n = 8–9/group). Mice were then euthanized followed by transcardial perfusion and tissue collection as described for the DIO and MC4R^−/−^ studies.

The PK11195 treatment experiment was performed as previously described by Gut and colleagues [19]. 8–10 week-old male mice were injected with vehicle (1.8% DMSO) or 5 mg/kg body weight PK11195 (Sigma-Aldrich, cat # C0424) at 8 a.m. and 2 p.m. Although animals do not usually consume much during the light period (7 am to 7 pm) food was withdrawn from the animals after the first injection to control for intake-associated variations in metabolic genes. Mice were euthanized at 4 p.m. and epididymal WAT collected and stored at −80°C until use.

### Histopathology and Immunohistochemistry (IHC) of Adipose Tissue

Epididymal WAT and interscapular BAT were dissected from DIO mice and fixed for 24 hours in neutral buffered formalin followed by paraffin embedding, sectioning, and hematoxylin and eosin (H&E) staining by the Translational Pathology Shared Resource at Vanderbilt University Medical Center. Sections were viewed and imaged using brightfield microscopy (AxioImager Z1, Zeiss, Thornwood, NY). For immunohistochemical staining, epididymal WAT from MC4R^−/−^ and their wild-type littermates (MC4R^+/+^) (n = 3/genotype) was fixed in 1% paraformaldehyde, as previously described [20]. Briefly, after fixation, adipose pieces were incubated in blocking solution (1∶5 Horse serum [Pel-Freeze Arkansas] in PBS +0.3% triton) for one hour followed by overnight incubation in primary antibodies diluted in blocking solution at 4°C. Primary antibodies used were raised against TSPO (1∶250, rabbit monoclonal, Cat # ab109497, Abcam, Cambridge, MA) and the macrophage marker F4/80 (1∶250, rat monoclonal, Cat # ab6640, Abcam, Cambridge, MA). The WAT was then incubated in fluorescence-conjugated secondary antibodies, Alexa 488 anti-rabbit and Alexa 568 anti-rat (1∶1000, Invitrogen, Eugene, OR). Imaging with laser confocal microscopy (Zeiss LSM 710, Carl Zeiss International, Germany) was performed in the Cell Imaging Shared Resource at Vanderbilt Medical Center. Images shown are representative of three independent fields examined from each animal.

### Real-time PCR

RNA was extracted from adipose tissue using Trizol (Invitrogen Inc., CA) followed by analysis on a NanoDrop ND-1000 spectrophotometer (ThermoScientific, Wilmington, DE) for concentration and purity determination. One microgram of RNA was digested with DNase (Promega, Madison, WI) to remove potential contaminating genomic DNA, followed by reverse transcription (iScript, Bio-Rad Inc., Hercules, CA) to synthesize cDNA according to manufacturer’s instructions. Quantitative real-time PCR was then performed using TaqMan Universal PCR Master mix (Applied Biosystems, Branchburg NJ) and TaqMan gene expression assay primer probes (Applied Biosystems, Foster City, CA) for TSPO (Mm00437828); peroxisome proliferator-activator receptor coactivator α (PGC1α; Mm00447183); F4/80 (Mm00802529); uncoupling protein 1 (UCP1; Mm01244861); sterol regulatory element binding transcription factor 1 (Srebf1; Mm00550338_m1); phosphoenolpyruvate carboxykinase (pck1; Mm00440636_m1); hormone sensitive lipase (Lipe; Mm00495359_m1); GAPDH (Mm99999915); and β-actin (Cat# 4352341E). A C1000 thermal cycler (CFX96 Real-Time System, Bio-Rad, Hercules, CA) in the Vanderbilt Molecular Cell Biology Resource Core was used with the following cycling conditions: 2 minutes at 50°C, 10 min at 95°C, followed by 40 cycles of 15 seconds at 95°C then 1 minute at 60°C. Cycle threshold values (Ct) values analyzed with CFX Bio-Rad software were used to determine relative quantification (RQ) values of gene expression for each gene by the ΔΔCt method. Actin or GAPDH were used as housekeeping genes. Due to limited amount of tissues, real-time PCR was performed on separate cohorts of lean (n = 10) and DIO (n = 9) mice than those used in binding studies.

### Preparation of Tissue Extracts for Ligand Binding Studies

Frozen WAT and BAT samples were homogenized in a 15 mL glass dounce homogenizer (Wheaton) on ice with 2.0 mL homogenizing buffer (final concentrations of 0.32 M Sucrose [Sigma-Aldrich, St. Louis, MO], 1 mM EDTA [Cellgro, Manassas, VA], 10 mM Tris-HCl pH 7.8, 1∶500 protease inhibitor cocktail [Sigma-Aldrich, St. Louis, MO]) per 200 mg tissue. A loose pestle (Wheaton) was used to break up the tissue followed by several strokes with the tight pestle (Wheaton). For whole lysate, 400 µL of homogenate was spun at 5,000 g at 4°C for 10 minutes followed by re-suspension of the pelleted material in homogenizing buffer and storage at −80°C. Mitochondrial extractions were performed on the remainder of the tissue homogenates based on methods previously reported in the literature [21]. The homogenate was spun at 1000 g for 10 minutes at 4°C to yield the first nuclear fraction (pelleted material). The supernatant was spun again at 1000 g for 10 minutes at 4°C to yield the second nuclear fraction. The supernatant was spun at 13000 g for 20 minutes at 4°C to yield the crude mitochondrial pellet. The resulting pellet was re-suspended in homogenizing buffer and spun at 13000 g for 10 minutes at 4°C resulting in the final mitochondrial fraction. Nuclear and mitochondrial fractions were re-suspended in homogenizing buffer and stored at −80°C. Protein concentrations were determined with Pierce BCA Protein assay kit (Thermoscientific, Rockford, IL) and absorbance at 562 nm analyzed by a plate reader (Spectramax M5, Molecular Devices, Sunnyvale, CA).

### TSPO Ligand Binding Assay to Determine Relative TSPO Expression

To assess relative TSPO protein expression, binding of ^3^H-PK11195 (specific activity = 85.7 Ci/mmol, PerkinElmer, Boston, MA) was quantified in mitochondrial extracts from obese mice as compared to lean controls. 20–40 µg of mitochondrial extracts diluted in 1X PBS (total volume 100 µL) were loaded into 24-well plates and allowed to incubate with 6 nM ^3^H-PK11195 for 2 hours at 4°C with gentle shaking. Bound fractions were then harvested by vacuum filtration through Whatman GF/B filter paper (FP100, Fisher Scientific, Hanover Park, IL) with a Brandel harvester (model M-24TI, Gaithersburg, MD). Filters were washed 5–6 times with 1X PBS, and then collected into vials containing 4 mL of scintillation fluid. Radioactive counts were determined by a Beckman LS 6500 scintillation counter (Beckman Coulter, Inc., Brea, CA). Counts in cpm were converted to fmol using the specific activity and scintillation counter efficiency, and then normalized by the amount of total protein loaded. Specific binding was determined by subtracting the nonspecific binding (binding in the presence of excess unlabeled PK11195 [10 µM, Sigma-Aldrich, St. Louis, MO]) from the total binding.

### Homologous Competition Binding Assay

Homologous competition binding assays (radioligand and unlabeled ligand are identical compounds) can be used in place of saturation binding assays to determine both affinity of the ligand for the binding site (as related by K_d_ value or equilibrium dissociation constant), and number of binding sites (Bmax) [22]. Assays were performed as described above but with ^3^H-PK11195 at a concentration of 1 nM incubated with varying concentrations of unlabeled PK11195 (10 pM –10 µM) to establish K_i_, (equilibrium dissociation constant) an indication of affinity, and Bmax. The generated curve established the IC_50_ concentrations (concentration of unlabeled drug at which half the specific binding is blocked) which could then be related to the K_i_ by the Cheng-Prussoff equation [23]. Since our competition binding assays were homologous (the radioligand and the cold ligand were tritiated and non-tritated versions of the same compound), the assumption can be made that the radioligand and unlabeled ligand have equal affinities, and thus, the K_i_ can be related to the K_d._

### Statistical Analysis

All data were expressed as means ± standard error of the mean (s.e.m.). Differences between lean and obese groups were analyzed by unpaired *t*-test using GraphPad Prism version 5.4 software (GraphPad software, San Diego, CA). Statistical significance was considered to be *p*<0.05, and is indicated on each figure accordingly. Competition curves were analyzed using a one-site homologous recombination model using GraphPad Prism version 5.4 software (GraphPad software, San Diego, CA).

## Results

### TSPO Gene Expression and Ligand Binding are Decreased by Obesity in White and Brown Adipose Tissue

**Figure 1:**
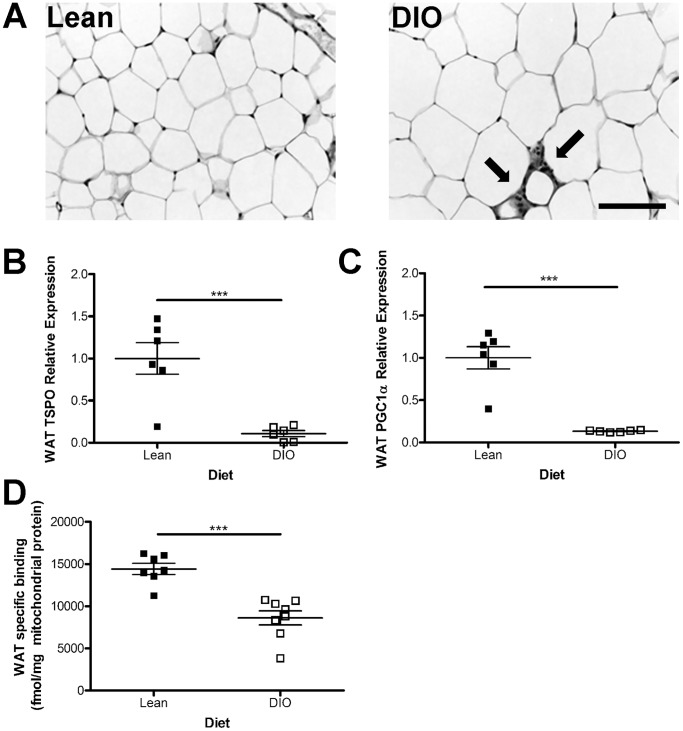
TSPO is down-regulated by diet-induced obesity in white adipose tissue. A) H&E staining of white adipose tissue (WAT) from lean and diet-induced obese (DIO) mice revealed larger, hypertrophic adipocytes in obese tissue surrounded by “crowning” macrophages (indicated by arrows). B) TSPO mRNA was significantly lower in DIO WAT than controls as was PGC1α (C), a positive control for mitochondrial dysfunction. D) Binding of TSPO ligand ^3^H-PK11195 in WAT mitochondrial extracts revealed significantly lower expression of TSPO binding sites in DIO mice as compared to lean controls. Data are expressed as mean ± standard error. n = 6–8. Statistical significance of ***p≤0.001 was determined by unpaired t-test. Scale bar = 100 µm.

After 19 weeks on a HFD, DIO mice had significantly increased total body weight (35% increase; *p*≤0.0001), fat mass (86% increase; *p*≤0.0001), and to a lesser extent muscle mass (12% increase; *p* = 0.0005), as compared to their lean controls (Table 1). Using H&E staining we confirmed that WAT from DIO mice had hypertrophic adipocytes (Fig. 1A, right panel) compared to lean controls (Fig. 1A, left panel). Furthermore, we saw the presence of macrophage crowning structures [24], around smaller adipocytes in the DIO animals as indicated by the arrows in Fig. 1A.

Considering that TSPO is critical for mitochondrial function, we hypothesized that TSPO expression would also be altered as a result of the known mitochondrial dysfunction in DIO WAT. Using quantitative real-time PCR we measured *TSPO* mRNA levels in whole WAT and found a 90% reduction in DIO animals compared to the lean controls (Fig. 1B; *p*≤0.001). As peroxisome proliferator-activator receptor coactivator (PGC1α) strongly induces mitochondrial biogenesis [25], we measured its gene expression in adipose tissue as a positive control for mitochondrial dysfunction. In agreement with published literature [26], our data shows that *PGC1α* gene expression decreased by 87% in whole WAT from DIO mice (Fig. 1C; p≤0.001) as compared to lean standard chow fed controls. To determine whether the decrease in *TSPO* gene expression was merely a function of reduced mitochondrial number, we next performed TSPO ligand binding assays on WAT mitochondrial extracts with ^3^H-PK11195 to determine the relative number of TSPO ligand binding sites. Nonspecific binding in the presence of excess unlabeled PK11195 (10 µM) was 25% of the total binding and was subtracted to obtain the specific binding. The average specific binding of ^3^H-PK11195 in DIO WAT was 40% lower than the lean standard chow controls (Fig. 1D; *p*≤0.001) suggesting a change in the number of TSPO ligand binding sites on the mitochondrial membrane.

**Figure 2:**
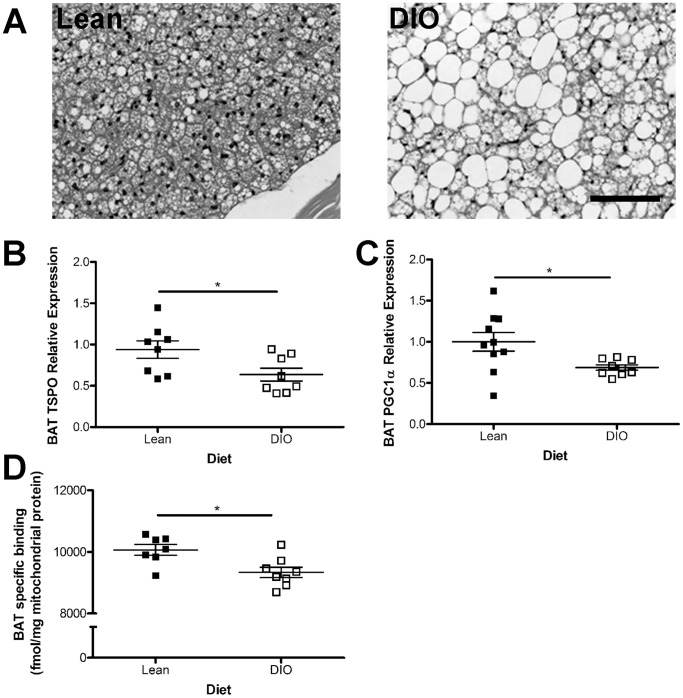
TSPO is down-regulated by diet-induced obesity in brown adipose tissue. A) H&E staining of brown adipose tissue (BAT) from diet-induced obese (DIO) mice revealed increased fat storage and hypertrophic adipocytes as compared to lean controls. B) TSPO mRNA was significantly lower in DIO BAT than controls as was PGC1α (C). D) Binding of TSPO ligand ^3^H-PK11195 in BAT mitochondrial extracts revealed significantly lower expression of TSPO binding sites in DIO mice as compared to controls. Data are expressed as mean ± standard error. n = 7–10. Statistical significance of *p<0.05 was determined by unpaired t-test. Scale bar = 100 µm.

Brown adipose tissue, the more thermogenic and mitochondrial dense of the two adipose tissues, releases energy as heat via the action of uncoupling protein-1 (UCP1) which uncouples oxidative phosphorylation from ATP synthesis [27]. Like WAT, BAT is rendered dysfunctional in obesity with enlarged adipocytes and increased lipid droplet size [28]) which we confirmed using H&E staining in our cohort of DIO mice (Fig. 2A). As seen in WAT from the same animals, *TSPO* gene expression in BAT was decreased by 32% (Fig. 2B, *p*<0.05) compared to controls on standard chow. Mitochondrial biogenesis was also reduced in BAT of DIO mice compared to standard chow controls as demonstrated by a 31% reduction in *PGC1α* gene expression (Fig. 2C; p<0.05). The relative TSPO ligand binding performed with ^3^H-PK11195 on BAT mitochondrial extracts revealed a 7% decrease in specific binding in DIO tissues compared to controls (Fig. 2D; p<0.05). Nonspecific binding in the presence of excess unlabeled PK11195 (10 µM) was 5% of the total binding and was subtracted to obtain the specific binding.

**Figure 3:**
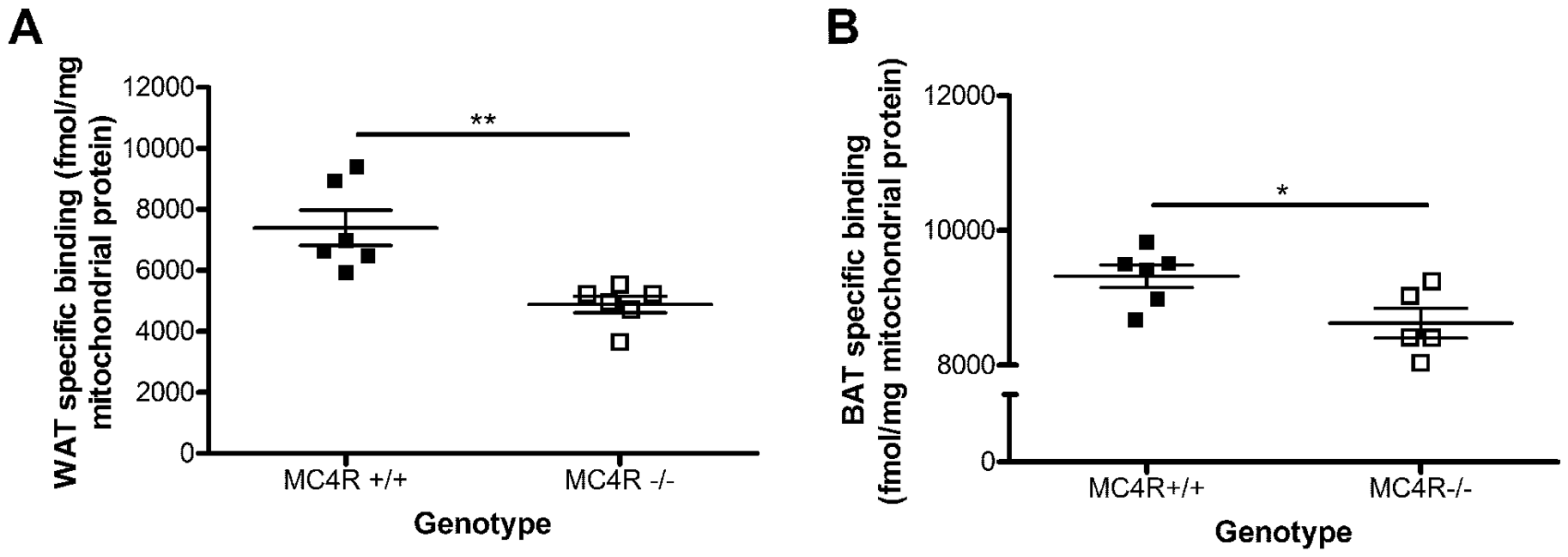
TSPO is down-regulated in white and brown adipose tissue of MC4R^−/−^ mice. Binding of TSPO ligand ^3^H-PK11195 in (A) white adipose tissue (WAT) and (B) brown adipose tissue (BAT) mitochondrial extracts revealed significantly lower expression of TSPO binding sites in MC4R^−/−^ mice as compared to wild-type littermate controls (MC4R^+/+^). Data are expressed as mean ± standard error. n = 5–6. Statistical significance of *p<0.05 and **p<0.01 was determined by unpaired t-test.

To determine whether this regulation of TSPO expression within mitochondria is specific to diet-induced obesity, we measured relative TSPO ligand binding using ^3^H-PK11195 in mitochondrial extracts from WAT and BAT from MC4R^−/−^ mice, a genetic model of obesity, as compared to their wild-type littermate controls. At 34 weeks, MC4R^−/−^ mice had significant increases in total body weight (45% increase; *p*<0.0001), fat mass (86% increase; *p*<0.0001), and muscle mass (29%; *p*<0.0001) compared with wild-type (MC4R^+/+^) littermates (Table 1). As seen in the DIO mice, MC4R^−/−^ animals had significantly lower levels of ^3^H-PK11195 binding in both WAT (34% decrease, Fig. 3A; *p*<0.01) and BAT mitochondrial extracts (9% decrease, Fig. 3B; *p*<0.05), though the difference was not as pronounced as in the DIO model.

### Obesity is Associated with Increased TSPO Expression in White Adipose Tissue Macrophages

**Figure 4:**
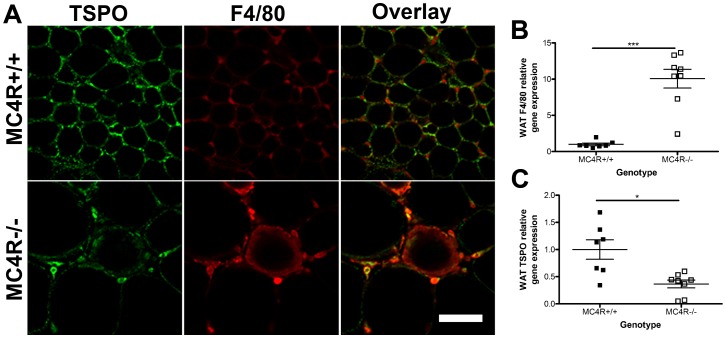
TSPO is expressed in adipocytes and white adipose tissue macrophages. TSPO (green) and macrophage marker F4/80 (red) co-localize in cells surrounding adipocytes in MC4R^−/−^ WAT (Overlay, bottom panel). F4/80 mRNA levels were significantly higher (B) and TSPO significantly lower (C) in MC4R^−/−^ mice as compared to their lean MC4R^+/+^ littermates. Data are expressed as mean ± standard error (panels B and C). n = 7–8 (panels B and C). Statistical significance of *p<0.05 and ***p<0.001 was determined by unpaired t-test. Scale bar = 50 µm.

We next sought to determine which cell types within the adipose tissue expressed TSPO and whether this expression pattern is altered as a function of obesity. We thus employed immunohistochemistry on whole pieces of fixed WAT from MC4R^−/−^ mice and wild-type littermate controls (MC4R^+/+^; Fig. 4A). TSPO immunoreactivity (green) was seen in adipocytes in both lean MC4R^+/+^ and their obese MC4R^−/−^ littermates. In the obese MC4R^−/−^ animals TSPO-immunoreactivity (green; bottom panel) was also seen in F4/80 immunoreactive cells (red) forming “crown-like” structures surrounding adipocytes (merged image, bottom panel). The increased F4/80 immunoreactivity was confirmed by quantitative real-time PCR data demonstrating a statistically significant 10-fold increase in F4/80 gene expression (*p*≤0.001) in obese MC4R^−/−^ mice compared to lean MC4R^+/+^ controls (Fig. 4B). Also in agreement with DIO mice, and TSPO ligand binding data, MC4R^−/−^ mice had significantly lower levels of TSPO gene expression in whole WAT (63% decrease, Fig. 4C; *p*<0.05).

### Obesity Regulates the Total Number of TSPO Ligand Binding Sites but does not Alter Binding Affinity in Brown Adipose Tissue

**Figure 5:**
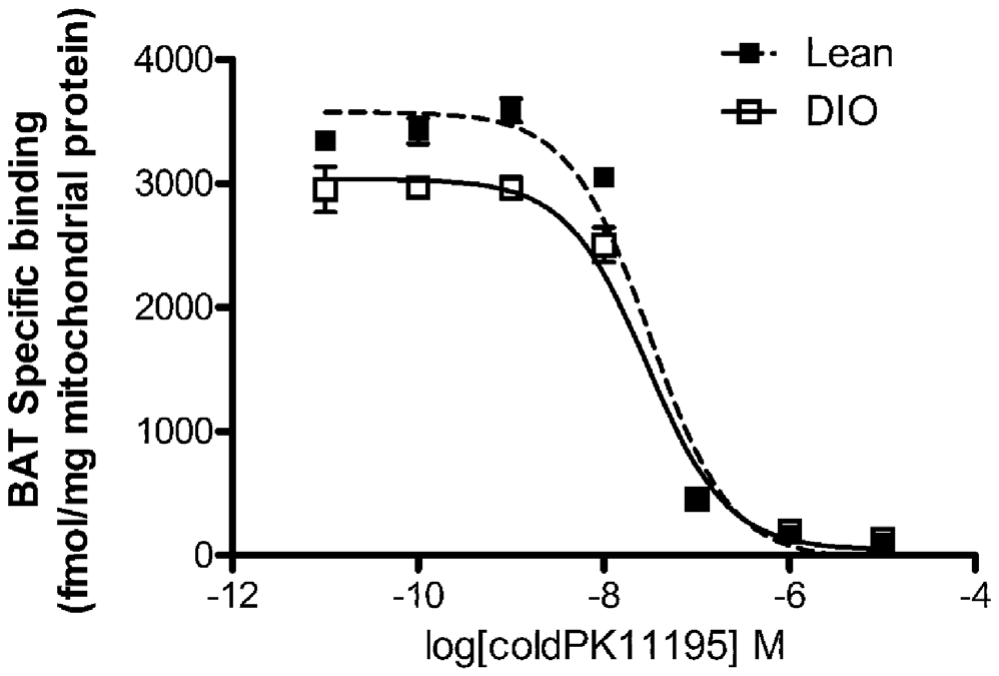
Diet-induced obesity is associated with a reduced number of TSPO ligand binding sites in brown adipose tissue. Homologous competition binding assay of TSPO ligand ^3^H-PK11195 with increasing concentrations of unlabeled PK11195 in brown adipose tissue (BAT) mitochondrial extracts shows a change in Bmax with no change in K_d_. n = 3 for each group.

We next performed competition binding assays to determine the total number of binding sites available (Bmax) and the affinity of the ligand for the receptor (K_d_) in BAT of DIO animals compared to lean controls (Fig. 5). In BAT from DIO mice, the K_d_ was not different from lean controls (DIO: 28.2 nM; lean 30.4 nM). However, there was a difference in the BAT Bmax between the two groups (DIO: 87292±18506 fmol/mg; lean: 113313±25032 fmol/mg protein). Unfortunately, due to the limited number of mitochondria that are obtained from WAT we were not able to perform the competition binding assay in this tissue.

### The Amount TSPO Ligand Binding is not Regulated in BAT by Acute Fasting or Cold Exposure

**Figure 6:**
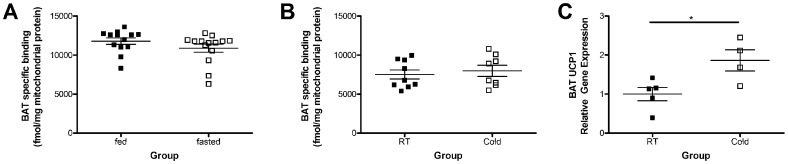
TSPO is not regulated in brown adipose tissue by fasting or cold exposure. Binding of TSPO ligand ^3^H-PK11195 in brown adipose tissue (BAT) mitochondrial extracts from mice fasted for 17 hours (A; n = 13–14) or exposed to 4–8°C for 4 hours (B; n = 8–9) revealed no significant differences in specific binding when compared to the fed (*p* = 0.1853) or room temperature (RT; *p* = 0.6163) control animals for each group respectively, as determined by unpaired t-test. BAT UCP1 mRNA was significantly increased in mice exposed to 4–8°C for 4 hours as compared to their control animals maintained at room temperature (C; n = 5/group). Data are expressed as mean ± standard error. *p<0.05 was determined by unpaired t-test.

Following the observation that TSPO is down-regulated by obesity in BAT mitochondria (Figs. 2 and 3), we next questioned whether acute metabolic challenges could affect TSPO expression. There was no significant difference in relative TSPO ligand binding in fasted mice compared to BAT from fed controls (Fig. 6A; *p = *0.1853). Similarly, there was also no significant difference in relative TSPO ligand binding in BAT from mice exposed to cold temperatures for 4 hours as compared to controls housed at room temperature (Fig. 6B; *p* = 0.6163). Exposure of mice to cold temperatures for 4 hours was sufficient to increase gene expression of uncoupling protein 1 (UCP1) in BAT (Fig. 6C).

### TSPO Ligand PK11195 Alters Expression of Metabolic Genes in WAT

**Figure 7:**
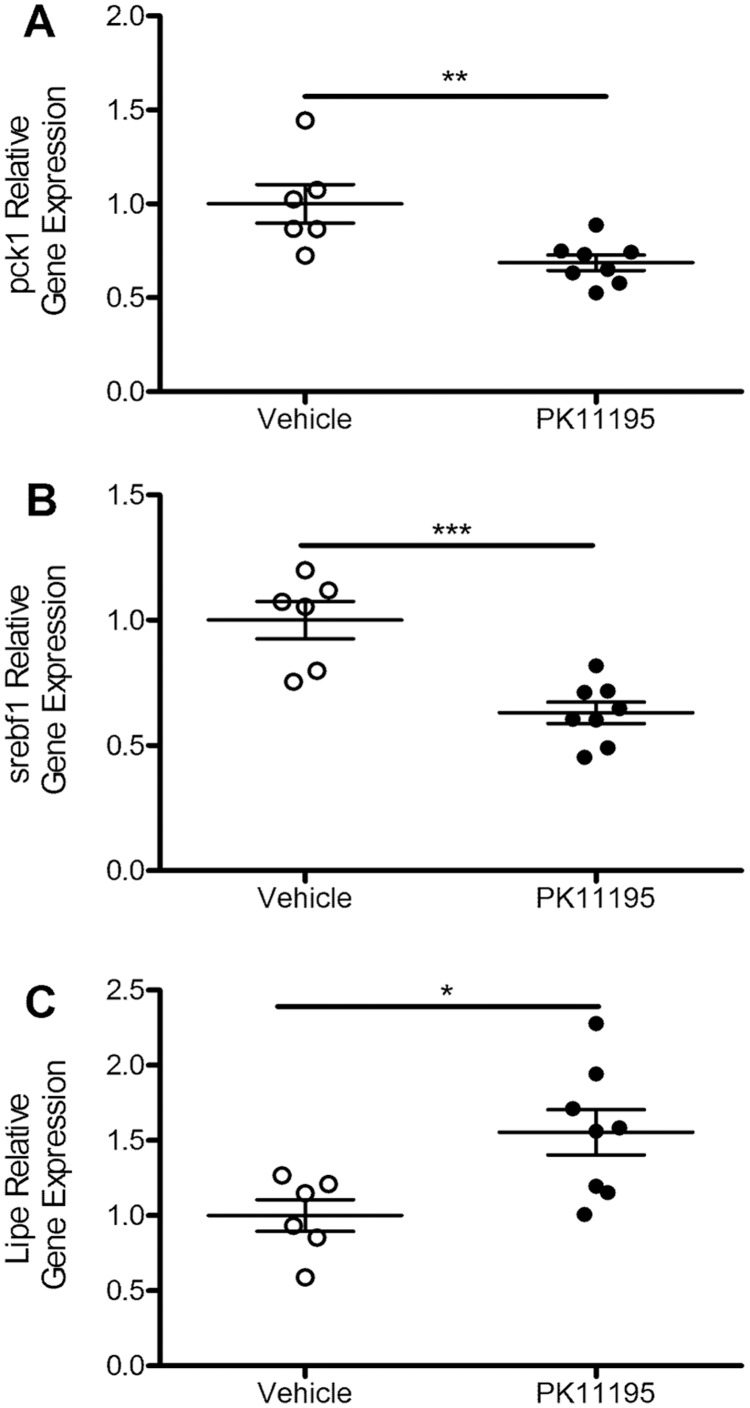
TSPO ligand PK11195 treatment alters mouse WAT metabolic gene expression. Intraperitoneal administration of PK11195 (5 mg/kg) in 8–10 week-old male C57BL/6 mice significantly decreased the expression of *pck1* (A) and *srebf1* (B) genes in WAT and significantly increased the *lipe* gene expression (C) as compared to vehicle-treated controls. Data are expressed as mean ± standard error. n = 6–8 for each treatment group. Statistical significance of *p<0.05, **p<0.01, and ***p<0.001 was determined by unpaired t-test.

It was recently demonstrated by Gut and colleagues [19] that treatment with the TSPO ligand PK11195 induces transcriptional changes in metabolic genes in the mouse liver. Following the same treatment paradigm as described in those studies, we evaluated transcriptional changes in genes involved in metabolism in WAT after administration of vehicle or PK11195 (5 mg/kg body weight). We observed a 31% decrease in the expression of the gene which encodes phosphoenolpyruvate carboxykinase (PEPCK; *pck1)* in WAT from PK11195-treated animals as compared to their vehicle-treated controls (Fig. 7A; *p* = 0.0084). In PK11195-treated animals, we also observed a 37% decrease in *srebf1* which encodes for sterol regulatory element-binding protein 1 (SREBP1; Fig. 7B; *p* = 0.0006), as well as a 55% increase in the hormone sensitive lipase (HSL; Fig. 7C; *p* = 0.0163) gene *lipe*.

## Discussion

Previously named the peripheral benzodiazepine receptor (PBR) for its ability to bind certain benzodiazepines in peripheral tissues, TSPO sits predominantly in the outer-mitochondrial membrane [29]. TSPO has been implicated in several vital mitochondrial processes including: i) binding to the voltage-dependent anion channel (VDAC) and adenine nucleotide translocase (ANT) as part of the mitochondrial permeability transition pore (mPTP; [30]); ii) regulation of ROS formation and apoptosis [31], [32]; and iii) regulation of cellular respiration and energy production [33], [34]. Perhaps the most well studied function of TSPO is uptake of cholesterol into mitochondria which is a critical step in steroid production [7], [34].

Here, for the first time, we have provided evidence that diet-induced obesity reduces TSPO in WAT and BAT on the gene expression level in whole tissue (Figs. 1B and 2B), as well as on the protein expression level within mitochondrial extracts as demonstrated by ligand binding studies (Figs. 1D and 2D). The reduction in TSPO expression does not merely reflect the decrease in mitochondrial biogenesis associated with obesity (Figs. 1C and 2C), as the ligand binding assays were performed on mitochondrial extracts and normalized to the total amount of protein loaded. Furthermore, the reduction in TSPO expression was not a direct result of the exposure to high-fat diet *per se* because we were able to detect a decrease in TSPO levels in mitochondrial extracts from WAT and BAT from the MC4R^−/−^ monogenic mouse model of obesity which were maintained standard chow (Figs. 3 and 4C).

As adipose tissue is heterogeneous we used immunohistochemistry to examine the distribution of TSPO-immunoreactivity. TSPO-immunoreactivity was seen in both adipocytes and F4/80-positive immune cells. The co-localization of TSPO and macrophage marker F4/80 in crown-like structures [24] of obese white adipose tissue (Fig. 4) is likely to reflect the increased phagocytic activity of these cells as TSPO is known to be up-regulated in macrophages and microglia in multiple inflammatory conditions [8], [9].

With an ever growing focus on BAT in the literature, and the factors that govern thermogenesis being of therapeutic interest [27], we next questioned whether or not TSPO could be regulated in BAT by other acute metabolic challenges. We subjected mice to either fasting or cold exposure, two processes which are known to decrease and increase BAT thermogenesis, respectively [35], [36]. Interestingly, we did not observe any significant changes in TSPO ligand binding in BAT from mice after a 17 hour fast (Fig. 6A) or 4 hours of cold exposure (Fig. 6B). In agreement with previously published data [37], we confirmed using gene expression analysis that 4 hours of cold exposure was sufficient to increase UCP1 expression in BAT (Fig. 6C) of cold exposed animals compared to controls housed at room temperature. These findings are in agreement with an earlier study using Ro-5-4864 which demonstrated that 4 hours of acute cold exposure did not regulate TSPO in BAT [13]. However, it is worth noting that one other study of chronic cold exposure treatment (15 days) in rats did report a decrease in TSPO levels in BAT using the radioligand benzodiazepine ^3^H-flunitrazepam [38], suggesting that chronic exposure to metabolic stressors may be required to regulate TSPO expression in BAT. Though it has been shown that UCP1 is not a critical component of the mitochondrial transition pore [39], to our knowledge, no one has specifically examined whether or not UCP1 has the ability to interact with or regulate the function of TSPO which would be very interesting given their shared location in the outer mitochondrial membrane.

The exact mechanism(s) through which TSPO is being down-regulated in obese mice remains uncertain. It is well known that obese humans often have elevated levels of serum cholesterol [40] which has also been observed in several mouse models of obesity including DIO rodents fed either a HFD [41] or HFHC diet [12], as well as in MC4R^−/−^ mice [42]. Furthermore, cholesterol imbalance in WAT is also a feature of obesity; a finding characterized by a dysregulation of cholesterol uptake and efflux within white adipocytes, which results in storage of free cholesterol inside lipid droplets [43]. In enlarged white adipocytes associated with obesity (as seen in Fig. 1A) cellular cholesterol increases with the size of the cell [43]. Activated BAT has the ability to burn stored lipids for energy [44]; however, in obesity brown adipocytes undergo a switch from multilocular lipid storage to unilocular storage more characteristic of white adipocytes (Fig. 2A; [28]). This increase in cholesterol storage in hypertrophic adipocytes in obesity may contribute to the reduction in TSPO documented in our study.

Two other factors associated with obesity-induced disease pathology that may also contribute to the regulation of TSPO in adipose tissue are oxidative stress and mitochondrial dysfunction. Similar to our observations in adipose tissue, a study by Dimitrova-Shumkovska et al [12] showed that rats placed on a HFHC diet have decreased TSPO expression in the liver and aorta, which correlated with increased oxidative stress in these tissues and systemic hypercholesterolemia. Moreover, it has been suggested that low levels of ROS increase TSPO expression while high levels of ROS decrease TSPO expression contributing to cell death [6]. Thus, while we have not directly measured ROS in our mice, we can speculate that adipose TSPO expression may be reduced by the high levels of ROS that have been previously demonstrated to be a feature of adipose tissue in obese animals [45].

Recent work by Gut et al [19] indicates that treatment with the TSPO ligand PK11195 in mice increases liver expression of the *pck1* gene which encodes the key metabolic enzyme PEPCK. Here, using the same experimental paradigm, we provide data showing that mice treated with PK11195 have reduced *pck1* and *srebf1* gene expression, and increased *lipe* expression in WAT compared to vehicle-treated controls (Fig. 7). Glucocorticoids have previously been shown to regulate *pck1* expression in a tissue specific manner; stimulating synthesis in liver and inhibiting synthesis in WAT [46]–[47]. Furthermore, the synthetic glucocorticoid dexamethasone increases HSL (the protein encoded by *lipe*) in adipocytes [48]. PK11195 is known to stimulate the hypothalamic-pituitary-adrenal (HPA) axis in rats [49], resulting in increases in plasma corticosterone. This may contribute observed increase in *lipe* and to the differential regulation of *pck1* observed between liver [19] and WAT; however, direct regulation at the cellular level cannot be ruled out. The decrease in WAT *srebf1* gene expression may reflect changes in WAT cholesterol metabolism as a result of modulating cholesterol uptake by TSPO through PK11195 administration.

In summary, our work demonstrates that TSPO is down-regulated in adipose tissue obesity but not by acute metabolic perturbations associated with fasting or cold-exposure. This combined with effects of the TSPO ligand PK11195 on the expression of metabolic genes in WAT provides important initial evidence that TSPO may be a potential therapeutic target for Metabolic Syndrome.

## References

[1] Ogden CL, Carroll MD, Kit BK, Flegal KM (2012) Prevalence of Obesity in the United States, 2009–2010. Centers for Disease Control and Prevention, National Center for Health Statistics. Available: http://www.cdc.gov/nchs/data/databriefs/db82.pdf. Accessed May 2013.

[2] Civitarese AE, Smith SR, Ravussin E (2007) Diet, energy metabolism, and mitochondrial biogenesis. Curr Opin Clin Nutr Metab Care 10: 679–687. 18089947 10.1097/MCO.0b013e3282f0ecd2

[3] Monteiro R, Azevedo I (2010) Chronic inflammation in obesity and the metabolic syndrome. Mediators Inflamm doi: 10.1155/2010/289645. (epub July 14, 2010). 10.1155/2010/289645 PMC2913796 20706689

[4] Bournat JC, Brown CW (2010) Mitochondrial dysfunction in obesity. Curr Opin Endocrinol Diabetes Obes 17: 446–452. 20585248 10.1097/MED.0b013e32833c3026 PMC5001554

[5] Xu H, Barnes GT, Yang Q, Tan G, Yang D, et al (2003) Chronic inflammation in fat plays a crucial role in the development of obesity-related insulin resistance. J Clin Invest 112: 1821–1830. 14679177 10.1172/JCI19451 PMC296998

[6] Batarseh A, Papadopoulos V (2010) Regulation of translocator protein 18 kDa (TSPO) expression in health and diseases states. Mol Cell Endocrinol 327: 1–12. 20600583 10.1016/j.mce.2010.06.013 PMC2922062

[7] Papadopoulos V, Amri H, Boujrad N, Cascio C, Culty M, et al (1997) Peripheral benzodiazepine receptor in cholesterol transport and steroidogenesis. Steroids 62: 21–28. 9029710 10.1016/s0039-128x(96)00154-7

[8] Venneti S, Lopresti BJ, Wiley CA (2006) The peripheral benzodiazepine receptor (Translocator protein 18 kDa) in microglia: from pathology to imaging. Prog Neurobiol 80: 308–322. 17156911 10.1016/j.pneurobio.2006.10.002 PMC1849976

[9] van der Laken CJ, Elzinga EH, Kropholler MA, Molthoff CF, van der Heijden JW, et al (2008) Noninvasive imaging of macrophages in rheumatoid synovitis using 11C-(R)-PK11195 and positron emission tomography. Arthritis Rheum 58: 3350–3355. 18975347 10.1002/art.23955

[10] Buck JR, McKinley ET, Hight MR, Fu A, Tang D, et al (2011) Quantitative, Preclinical PET of Translocator Protein Expression in Glioma Using 18F-N-Fluoroacetyl-N-(2,5-Dimethoxybenzyl)-2-Phenoxyaniline. J Nucl Med 52: 107–114. 21149488 10.2967/jnumed.110.081703 PMC3027353

[11] Owen DR, Matthews PM (2011) Imaging brain microglial activation using positron emission tomography and translocator protein-specific radioligands. Int Rev Neurobiol 101: 19–39. 22050847 10.1016/B978-0-12-387718-5.00002-X

[12] Dimitrova-Shumkovska J, Veenman L, Ristoski T, Leschiner S, Gavish M (2010) Chronic high fat, high cholesterol supplementation decreases 18 kDa Translocator Protein binding capacity in association with increased oxidative stress in rat liver and aorta. Food Chem Toxicol 48: 910–921. 20060027 10.1016/j.fct.2009.12.032

[13] Hirsch JD (1984) Pharmacological and physiological properties of benzodiazepine binding sites in rodent brown adipose tissue. Comp Biochem Physiol C 77: 339–343. 6144440 10.1016/0742-8413(84)90025-2

[14] Wang HJ, Fan J, Papadopoulos V (2012) Translocator protein (Tspo) gene promoter-driven green fluorescent protein synthesis in transgenic mice: an in vivo model to study Tspo transcription. Cell Tissue Res 350: 261–275. 22868914 10.1007/s00441-012-1478-5 PMC3740157

[15] Campioli E, Batarseh A, Li J, Papadopoulos V (2011) The endocrine disruptor mono-(2-ethylhexyl) phthalate affects the differentiation of human liposarcoma cells (SW 872). PLoS ONE 6: e28750 doi:10.1371/journal.pone.0028750 22205965 10.1371/journal.pone.0028750 PMC3244402

[16] Wade FM, Wakade C, Mahesh VB, Brann DW (2005) Differential expression of the peripheral benzodiazepine receptor and gremlin during adipogenesis. Obes Res. 13: 818–822. 10.1038/oby.2005.93 15919833

[17] Campioli E, Carnevale G, Avallone R, Guerra D, Baraldi M (2011) Morphological and receptorial changes in the epididymal adipose tissue of rats subjected to a stressful stimulus. Obesity (Silver Spring) 19: 703–708. 20948513 10.1038/oby.2010.244

[18] Huszar D, Lynch CA, Fairchild-Huntress V, Dunmore JH, Fang Q, et al (1997) Targeted disruption of the melanocortin-4 receptor results in obesity in mice. Cell 88: 131–141. 9019399 10.1016/s0092-8674(00)81865-6

[19] Gut P, Baeza-Raja B, Andersson O, Hasenkamp L, Hsiao J, et al (2013) Whole-organism screening for gluconeogenesis identifies activators of fasting metabolism. Nat Chem Biol 9: 97–104. 23201900 10.1038/nchembio.1136 PMC3552031

[20] Lumeng CN, DelProposto JB, Westcott DJ, Saltiel AR (2008) Phenotypic switching of adipose tissue macrophages with obesity is generated by spatiotemporal differences in macrophage subtypes. Diabetes 57: 3239–3246. 18829989 10.2337/db08-0872 PMC2584129

[21] Wieckowski MR, Giorgi C, Lebiedzinska M, Duszynski J, Pinton P (2009) Isolation of mitochondria-associated membranes and mitochondria from animal tissues and cells. Nat Protoc 4: 1582–1590. 19816421 10.1038/nprot.2009.151

[22] Wiener HL, Reith ME (1992) Determination of radioligand specific activity using competition binding assays. Anal Biochem 207: 58–62. 1489100 10.1016/0003-2697(92)90499-w

[23] Cheng Y, Prusoff WH (1973) Relationship between the inhibition constant (KI) and the concentration of inhibitor which causes 50 per cent inhibition (I50) of an enzymatic reaction. Biochem Pharmacol 23: 3099–3108. 10.1016/0006-2952(73)90196-2 4202581

[24] Cinti S, Mitchell G, Barbatelli G, Murano I, Ceresi E, et al (2005) Adipocyte death defines macrophage localization and function in adipose tissue of obese mice and humans. J Lipid Res 46: 2347–2355. 16150820 10.1194/jlr.M500294-JLR200

[25] Scarpulla RC (2008) Transcriptional paradigms in mammalian mitochondrial biogenesis and function. Physiol Rev. 88: 611–638. 10.1152/physrev.00025.2007 18391175

[26] Semple RK, Crowley VC, Sewter CP, Laudes M, Christodoulides C, et al (2004) Expression of the thermogenic nuclear hormone receptor coactivator PGC-1alpha is reduced in the adipose tissue of morbidly obese subjects. Int J Obes Relat Metab Disord 28: 176–179. 14557831 10.1038/sj.ijo.0802482

[27] Stephens M, Ludgate M, Rees DA (2011) Brown fat and obesity: the next big thing? Clin Endocrinol (Oxf) 74: 661–670. 21521287 10.1111/j.1365-2265.2011.04018.x

[28] Cinti S (2011) Between brown and white: novel aspects of adipocyte differentiation. Ann Med 43: 104–115. 21254898 10.3109/07853890.2010.535557

[29] Papadopoulos V, Baraldi M, Guilarte TR, Knudsen TB, Lacapère JJ, et al (2006) Translocator protein (18 kDa): new nomenclature for the peripheral-type benzodiazepine receptor based on its structure and molecular function. Trends Pharmacol Sci 27: 402–409. 16822554 10.1016/j.tips.2006.06.005

[30] McEnery MW, Snowman AM, Trifiletti RR, Snyder SH (1992) Isolation of the mitochondrial benzodiazepine receptor: association with the voltage-dependent anion channel and the adenine nucleotide carrier. Proc Natl Acad Sci U S A 89: 3170–3174. 1373486 10.1073/pnas.89.8.3170 PMC48827

[31] Carayon P, Portier M, Dussossoy D, Bord A, Petitprêtre G, et al (1996) Involvement of peripheral benzodiazepine receptors in the protection of hematopoietic cells against oxygen radical damage. Blood 87: 3170–3178. 8605331

[32] Veenman L, Papadopoulos V, Gavish M (2007) Channel-like functions of the 18-kDa translocator protein (TSPO): regulation of apoptosis and steroidogenesis as part of the host-defense response. Curr Pharm Des 13: 2385–2405. 17692008 10.2174/138161207781368710

[33] Hirsch JD, Beyer CF, Malkowitz L, Beer B, Blume AJ (1989) Mitochondrial benzodiazepine receptors mediate inhibition of mitochondrial respiratory control. Mol Pharmacol 35: 157–163. 2464128

[34] Lacapère JJ, Papadopoulos V (2003) Peripheral-type benzodiazepine receptor: structure and function of a cholesterol-binding protein in steroid and bile acid biosynthesis. Steroids 68: 569–585. 12957662 10.1016/s0039-128x(03)00101-6

[35] Boss O, Samec S, Dulloo A, Seydoux J, Muzzin P, et al (1997) Tissue-dependent upregulation of rat uncoupling protein-2 expression in response to fasting or cold. FEBS Lett. 412: 111–114. 10.1016/s0014-5793(97)00755-2 9257701

[36] Trayhurn P, Jennings G (1988) Nonshivering thermogenesis and the thermogenic capacity of brown fat in fasted and/or refed mice. Am J Physiol 254: R11–16. 2827526 10.1152/ajpregu.1988.254.1.R11

[37] Voss-Andreae A, Murphy JG, Ellacott KL, Stuart RC, Nillni EA, et al (2007) Role of the central melanocortin circuitry in adaptive thermogenesis of brown adipose tissue. Endocrinology 148: 1550–1560. 17194736 10.1210/en.2006-1389

[38] Gonzalez Solveyra C, Romeo HE, Rosenstein RE, Estevez AG, Cardinali DP (1988) Benzodiazepine binding sites in rat interscapular brown adipose tissue: effect of cold environment, denervation, and endocrine ablations. Life Sci 42: 393–402. 2828790 10.1016/0024-3205(88)90077-x

[39] Crichton PG, Parker N, Vidal-Puig AJ, Brand MD (2009) Not all mitochondrial carrier proteins support permeability transition pore formation: no involvement of uncoupling protein 1. Biosci Rep 30: 187–92. 19622065 10.1042/BSR20090063 PMC2805926

[40] Després JP, Moorjani S, Lupien PJ, Tremblay A, Nadeau A, et al (1990) Regional distribution of body fat, plasma lipoproteins, and cardiovascular disease. Arteriosclerosis 10: 497–511. 2196040 10.1161/01.atv.10.4.497

[41] Hoffler U, Hobbie K, Wilson R, Bai R, Rahman A, et al (2009) Diet-induced obesity is associated with hyperleptinemia, hyperinsulinemia, hepatic steatosis, and glomerulopathy in C57Bl/6J mice. Endocrine 36: 311–325. 19669948 10.1007/s12020-009-9224-9 PMC4219357

[42] Itoh M, Suganami T, Nakagawa N, Tanaka M, Yamamoto Y, et al (2011) Melanocortin 4 receptor-deficient mice as a novel mouse model of nonalcoholic steatohepatitis. Am J Pathol 179: 2454–2463. 21906580 10.1016/j.ajpath.2011.07.014 PMC3204024

[43] Le Lay S, Ferré P, Dugail I (2004) Adipocyte cholesterol balance in obesity. Biochem Soc Trans 32: 103–106. 14748723 10.1042/bst0320103

[44] Bartelt A, Merkel M, Heeren J (2012) A new, powerful player in lipoprotein metabolism: brown adipose tissue. J Mol Med (Berl) 90: 887–893. 22231746 10.1007/s00109-012-0858-3

[45] Furukawa S, Fujita T, Shimabukuro M, Iwaki M, Yamada Y, et al (2004) Increased oxidative stress in obesity and its impact on metabolic syndrome. J Clin Invest 114: 1752–1761. 15599400 10.1172/JCI21625 PMC535065

[46] Meyuhas O, Reshef L, Gunn JM, Hanson RW, Ballard FJ (1976) Regulation of phosphoenolpyruvate carboxykinase (GTP) in adipose tissue in vivo by glucocorticoids and insulin. Biochem J 158: 1–7. 962885 10.1042/bj1580001 PMC1163929

[47] Nechushtan H, Benvenisty N, Brandeis R, Reshef L (1987) Glucocorticoids control phosphoenolpyruvate carboxykinase gene expression in a tissue specific manner. Nucleic Acids Res 15: 6405–6417. 3627993 10.1093/nar/15.16.6405 PMC306113

[48] Xu C, He J, Jiang H, Zu L, Zhai W, et al (2009) Direct effect of glucocorticoids on lipolysis in adipocytes. Mol Endocrinol 23: 1161–1170. 19443609 10.1210/me.2008-0464 PMC5419195

[49] Calogero AE, Kamilaris TC, Bernardini R, Johnson EO, Chrousos GP, et al (1990) Effects of peripheral benzodiazepine receptor ligands on hypothalamic-pituitary-adrenal axis function in the rat. J Pharmacol Exp Ther 253: 729–737. 2160009

